# Enteric Infection with *Citrobacter rodentium* Induces Coagulative Liver Necrosis and Hepatic Inflammation Prior to Peak Infection and Colonic Disease

**DOI:** 10.1371/journal.pone.0033099

**Published:** 2012-03-09

**Authors:** Arkadiusz R. Raczynski, Sureshkumar Muthupalani, Katherine Schlieper, James G. Fox, Steven R. Tannenbaum, David B. Schauer

**Affiliations:** 1 Department of Biological Engineering, Massachusetts Institute of Technology, Cambridge, Massachusetts, United States of America; 2 Division of Comparative Medicine, Massachusetts Institute of Technology, Cambridge, Massachusetts, United States of America; French National Centre for Scientific Research, France

## Abstract

Acute and chronic forms of inflammation are known to affect liver responses and susceptibility to disease and injury. Furthermore, intestinal microbiota has been shown critical in mediating inflammatory host responses in various animal models. Using *C. rodentium*, a known enteric bacterial pathogen, we examined liver responses to gastrointestinal infection at various stages of disease pathogenesis. For the first time, to our knowledge, we show distinct liver pathology associated with enteric infection with *C. rodentium* in C57BL/6 mice, characterized by increased inflammation and hepatitis index scores as well as prominent periportal hepatocellular coagulative necrosis indicative of thrombotic ischemic injury in a subset of animals during the early course of *C. rodentium* pathogenesis. Histologic changes in the liver correlated with serum elevation of liver transaminases, systemic and liver resident cytokines, as well as signal transduction changes prior to peak bacterial colonization and colonic disease. *C. rodentium* infection in C57BL/6 mice provides a potentially useful model to study acute liver injury and inflammatory stress under conditions of gastrointestinal infection analogous to enteropathogenic *E. coli* infection in humans.

## Introduction

Liver responses under acute and chronic forms of inflammation have gained considerable interest, particularly due to the role of inflammation in alcoholic liver disease (ALD), non-alcoholic steatohepatitis (NASH) [1], [2], ischemia/reperfusion (I/R) injury [3], [4], and drug-induced liver injury (DILI) [5], [6]. The complexity of these responses is underscored by the liver's key role in innate immunity, providing initial defense against microbes, bacterial products, and toxins traversing the intestinal barrier [7], [8]. Furthermore, understanding the host response to environmental pathogens and chemicals is critical in order to study how exposure may amplify, synthesize with, or mitigate hepatic injury and disease. While genetic manipulation and pharmacological inhibition have facilitated our understanding of hepatic homeostasis under inflammatory stress conditions, there are few animal models that can reliably predict these pathological perturbations in humans.

*Citrobacter rodentium* (*C. rodentium*) is an enteric bacterial pathogen that causes varying degrees of intestinal inflammation, hyperplasia, and edema in numerous strains of mice [9], [10], [11]. As a murine homolog of enteropathogenic *Escherichia coli* (*E. coli*) (EPEC), *C. rodentium* infection in mice is widely used as an animal model to study these human infections; it provides a reproducible, robust, and physiologically relevant model of inflammation. More recently, *C. rodentium* infection has demonstrated organ-specific effects distal to the primary site of attachment and disease. These include alterations of phase I (cytochrome P450s) and phase II (glucuronosyltransferases (UGTs)) metabolic enzymes in liver and kidney, as well as increases in hepatic cytokine transcript [12], [13]; a time course of regulation that follows colonic inflammation and bacterial colonization, peaking at 7–10 days post inoculation (DPI), and returning to normal by 15–24 DPI. Changes in local and systemic cytokines have been implicated in metabolic dysregulation potentially altering host susceptibility to injury and disease [14]. Furthermore, altered phase I/II enzymes, which are associated with biosynthesis and catabolism of endogenous substrates, as well as clearance of numerous pharmaceuticals, could be of clinical significance for patients presenting with liver diseases, inflammatory bowel syndromes, or pathogenic gastrointestinal infections.

Here we examined the host response to *C. rodentium* at various stages in the course of enteric infection, focusing particularly on systemic and liver-specific cytokine protein profiles. For the first time, we show distinct liver pathology associated with enteric infection with *C. rodentium* in C57BL/6 mice, characterized by portal vein thrombi and associated periportal ischemic necrosis during the early stages of pathogenic infection (3 DPI) in a subset of animals. Hepatic injury and inflammation correlated with serum elevation of liver transaminases, systemic and liver resident cytokines, as well as signal transduction changes prior to peak *C. rodentium* colonization and colonic disease.

## Results

### Infection kinetics and *C. rodentium-induced* histological colonic changes

Although dependent on mouse strain, *C. rodentium* colonization levels in the colon typically peaks 5–14 DPI with approximately 10^9^ colony-forming units (CFU)/g feces [9]. As previously reported, fecal shedding of *C. rodentium* in C57BL/6 mice reached a maximum of ∼10^9^ CFU/g feces [15], with detectable levels 3 DPI, peaking 7 DPI, and clearance beginning as early as 10 DPI (**Figure S1A**). Body weight changes were not significant over the course of infection (**Figure S1B**); consistent with previous findings that *C. rodentium* infection in adult C57BL/6 mice results in self-limiting disease with minimal morbidity and mortality [9], [10], [16].

**Figure 1:**
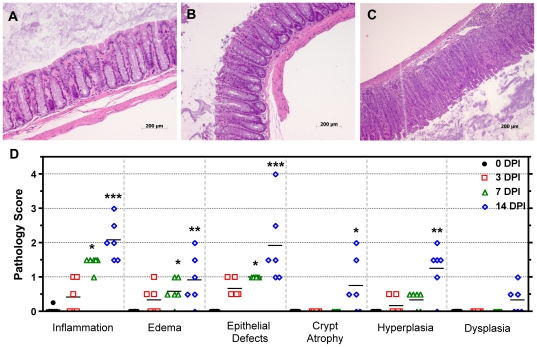
*C. rodentium*-induced colonic effects in C57BL/6 mice. (**A**) Mock inoculated animals at 0 DPI with normal colonic architecture where epithelial integrity and goblet cells appear intact. (**B**) Colon at 3 DPI showing epithelial defects at the top of the crypt. (**C**) Colon at 14 DPI demonstrating hyperplastic crypts and depletion of goblet cells. (**D**) *C. rodentium* induced statistically significant histological changes as early as 7 DPI (inflammation, edema, epithelial defects) in colonic sections and found to be most dramatic at 14 DPI. Crypt atrophy and minimal dysplastic changes were only noticeable at 14 DPI. Changes in inflammation, edema, epithelial defects, and hyperplasia as early as 3 DPI were noted, but failed to reach statistical significance (Kruskal-Wallis non-parametric test with Dunn's multiple comparison test: * P<0.05, ** P<0.01, *** P<0.001). Symbols indicate individual animals and lines indicate group means.

Histomorphological changes and disease severity scores of hematoxylin and eosin (H&E) stained sections of the ileo-cecal junction and colon were tabulated by a board certified veterinary pathologist (SM) blinded to study treatment groups (**Figure 1**). Intact epithelium with none to minimal changes were noted in the control animals; however in the infected group as early as 3 DPI, epithelial defects, colonic foci of inflammation, edema, and hyperplasia were observed (**Figure 1A and 1B**) with statistically significant increases in inflammation, edema, and epithelial defects by 7 DPI (P<0.05, Kruskal-Wallis with Dunn's post test), and reaching maximal severity by 14 DPI (**Figure 1C and 1D**). Significant changes in crypt atrophy and epithelial hyperplasia were only observed at 14 DPI (**Figure 1D**) (P<0.05 and P<0.01 respectively). In adult C57BL/6 mice, disease peaks approximately 2 weeks post inoculation (WPI) with recovery and full clearance of *C. rodentium* by 4 WPI and resolution of colonic lesions by 6 WPI [**9]**, [10], [17****].

### *C. rodentium*-induced liver inflammation and necrosis

**Figure 2:**
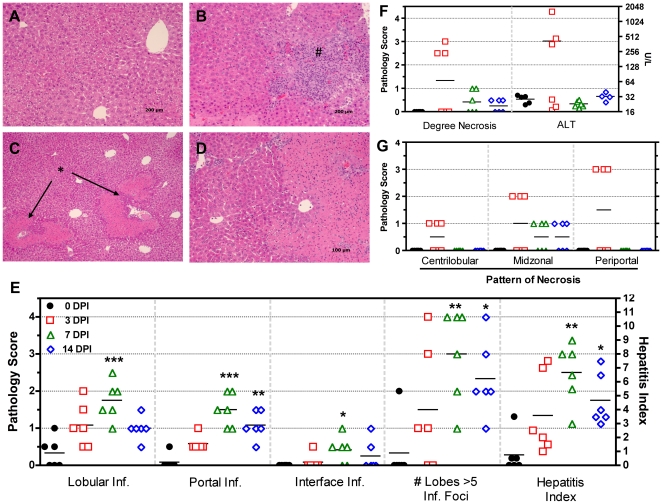
*C. rodentium*-induced necrosis and histological liver changes in C57BL/6 mice. (**A**) Control livers appeared normal with minimal observable histology. (**B**) At 3 DPI the appearance of a focus of lobular injury and inflammation indicating a pro-inflammatory state (indicated by **#**). (**C**) Multifocal venous thrombi and associated periportal hepatocellular coagulative necrosis (indicated by *) was observed at 3 DPI suggestive of thrombotic ischemic injury. (**D**) Higher magnification (400×) view showing necrotic hepatocytes with eosinophilic cytoplasm, appearance of pyknotic or absence of hepatic nuclei, and loss of normal cellular architecture. (**E**) *C. rodentium* induced histological changes as early as 3 DPI in liver sections, statistically significant at 7 DPI (portal, lobular, interface inflammation, # lobes with >5 inflammatory foci, and hepatitis index score), with moderate improvement by 14 DPI. (**F**) The degree of necrosis determined by pathological assessment as well as serum ALT measurements. (**G**) The pattern of necrosis was assessed as centrilobular, midzonal, or periportal in distribution. (Kruskal-Wallis with Dunn's post test compared to controls: * P<0.05, ** P<0.01, *** P<0.001). Symbols indicate individual animals and lines indicate group means.

While the effect of *C. rodentium* infection has been well characterized with respect to colonic changes, we decided to examine in greater detail the effect of *C. rodentium* infection on liver homeostasis. *C. rodentium* induced histological changes as early as 3 DPI in liver sections that reached statistical significance at 7 DPI (portal inflammation, lobular inflammation, interface inflammation, number of lobes with >5 inflammatory foci, and hepatitis index), with moderate improvement noted by 14 DPI (**Figure 2E**). The overall hepatitis index of 4 or more is a more convincing feature of true hepatitis and this was observed more often at later time points. Control livers had none to minimal observable histological abnormalities, however, in infected animals at 3 DPI, the appearance of foci of inflammation indicated a pro-inflammatory state with multifocal coagulative necrosis observed in 3/6 mice (**Figure 2A–D**). Necrotic lesions presented primarily with a periportal pattern of distribution (**Figure 2G**) and were indicative of thrombotic ischemic injury with the presence of portal venular fibrin thrombi. Necrotic regions contained hepatocytes with eosinophillic cytoplasms, appearance of pyknotic or absent nuclei, and loss of normal cellular architecture. To confirm and measure the extent of hepatic necrosis, serum was processed for alanine amino transferase (ALT), the levels of which are known to rapidly increase and persist subsequent to liver injury. Hepatic necrosis score correlated with increased ALT at 0 vs 3 DPI = (28.60±2.358 U L^−1^ N = 5 versus 415.2±250.4 U L^−1^ N = 6). The three mice with hepatocellular necrosis had ALT levels 56, 16, and 13-fold higher than average control values (ALT = 1603, 464, and 359 U L^−1^ respectively), while mice without these hepatic lesions at 3 DPI, had ALT levels comparable to 0 DPI controls (**Figure 2F**).

**Figure 3:**
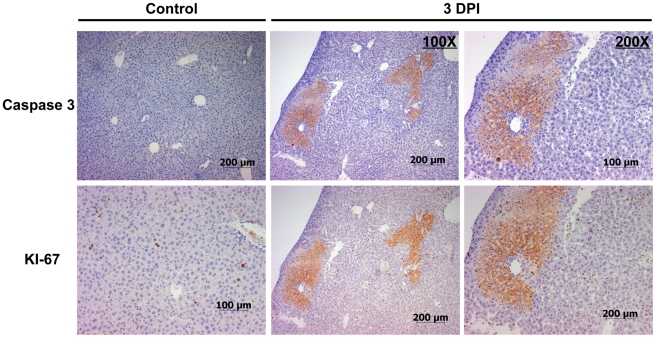
Activated caspase 3 and Ki-67 in *C. rodentium*-induced liver lesions. Control livers showed minimal staining for the apoptotic marker activated caspase 3, and proliferative marker Ki-67 (far left panels), while livers with necrosis at 3 DPI stained positively for both markers with a periportal pattern of distribution (middle (100×) and far right (200×)). Pictured is a representative liver section that was serially sectioned and immunostained independently for each cellular marker.

**Figure 4:**
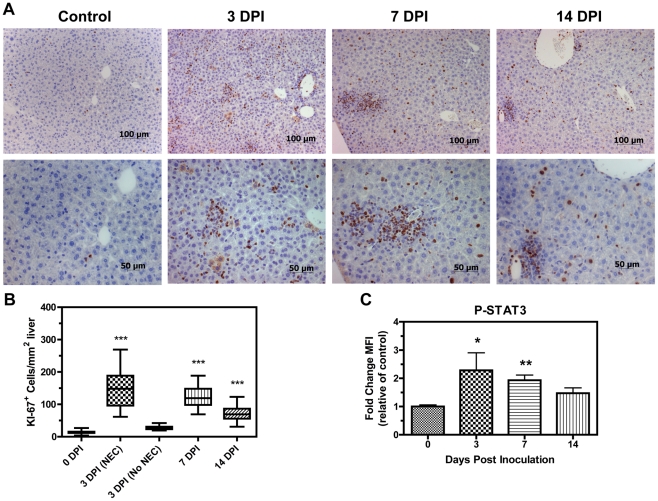
*C. rodentium* significantly increases Ki-67^+^ labeling index and STAT3 phosphorylation in livers. (**A**) Top panels are 200× and lower panels are 400× views of the same frame. Brown-colored pigment indicates positively stained cells for Ki-67. (**B**) Average number of Ki-67^+^ cells/mm^2^ liver determined in 15 fields (magnification, 400×, 3 fields/lobe) per mouse (*n* = 3 for 0, 7, and 14 DPI; *n* = 2 for 3 DPI (NEC); *n* = 1 for 3 DPI (No NEC)). Data is represented as box-whisker plots, where boxes represent the first to third quartile and a horizontal line indicates the median. Bars represent ranges. (One-way ANOVA with Tukey's multiple comparison test: *** P<0.001). (**C**) P-STAT3 levels in liver lysates were detected using a Bio-Rad phosphoprotein panel and expressed as a fold change of mean fluorescence intensity (MFI) relative to controls (0 DPI). STAT3 was significantly activated in livers of mice inoculated with *C. rodentium* at 3 and 7 DPI (Kruskal-Wallis test with Dunn's multiple comparisons test: * P<0.05, ** P<0.01).

Necrotic livers were further characterized by immunohistological staining for activated caspase 3 and Ki-67 and revealed increased labeling index of both markers in periportal areas of injury indicating heterogeneous regions of cell death and active proliferation (**Figure 3**). Livers harboring necrotic lesions at 3 DPI contained a statistically higher incidence of positively stained cells for Ki-67, as compared to controls and those without lesions at 3 DPI, indicating a proliferative state in these livers with a comparable increase in labeling index at 7 and 14 DPI (**Figure 4A and 4B**). Animals at 7 and 14 DPI did not harbor obvious necrotic lesions or elevations in ALT; however, the presence of large hepatocytes and areas of necro-granulomatous inflammation indicated that prior acute hepatic necrosis in these animals may have resolved.

### Serum cytokine and chemistry changes due to *C. rodentium* infection

*C. rodentium* has been shown to induce numerous immune regulators at both systemic and local tissue levels, and are generally associated with a mucosal Th1/Th17-mediated response in C57BL/6 mice [18], [19]. Recently, serum and colon matched cytokines were analyzed in *C. rodentium* infected C57BL/6 mice at peak colonic disease (14 DPI), demonstrating correlations of colon and systemic levels associated with disease severity [20]. Here we measured both serum-specific changes in cytokines/chemokines as well as serum chemistries at early, peak, and resolving timepoints of bacterial clearance (**Figures S2 and S3**). Numerous circulating cytokines and chemokines were significantly induced at 3, 7, and 14 DPI. Findings at 3 DPI were particularly interesting given that they correlated with histological findings in the liver and preceded peak colonic bacterial colonization and disease. Circulating cytokines were significantly elevated for IL-2, G-CSF, GM-CSF, MCP-1, MIP-1β, and RANTES at 3 DPI as a group (P<0.05, by one way ANOVA with Tukey's multiple comparison test). Elevations in IL-10 and KC were noted but did not reach statistical significance. For the majority of cytokines/chemokines measured, mice at 7 DPI appeared to have the highest circulating levels correlating with peak fecal *C. rodentium* shedding (**Figure S2**). This included elevations in pro-inflammatory (IL1β, IL-6, and TNF-α), immunomodulatory (IL-2, IL-12p70, IL-17), Th2 (IL-4, IFN-γ), and macrophage chemotaxis/activation (GM-CSF, MIP-1β) (P<0.001) when compared to 0 DPI controls.

Serum cholesterol levels also increased significantly at 3 DPI and continued to rise until 14 DPI. It has been shown that *C. rodentium* can alter the transcriptional levels of certain phase I/II metabolic enzymes in both liver and kidney associated with metabolism of endogenous and pharmaceutical substrates [12], [13]. Cholesterol's clearance from the circulation is partially mediated by oxidation via CYP 7A1, and we have shown decreases in CYP7a1 transcript in livers of mice infected with *C. rodentium* (data not shown). Significant increase in blood urea nitrogen (BUN) was noted at 14 DPI (P<0.01) as well as decreases in creatine phosphokinase (CPK) at 7 and 14 DPI (P<0.05 and P<0.01 respectively). Total bilirubin at 14 DPI was also decreased (P<0.01). Other serum chemistries and electrolytes measured, including albumin and glucose, were normal and did not vary significantly across monitored timepoints (**Figure S2**).

### Liver cytokine and signaling changes due to *C. rodentium* infection

**Figure 5:**
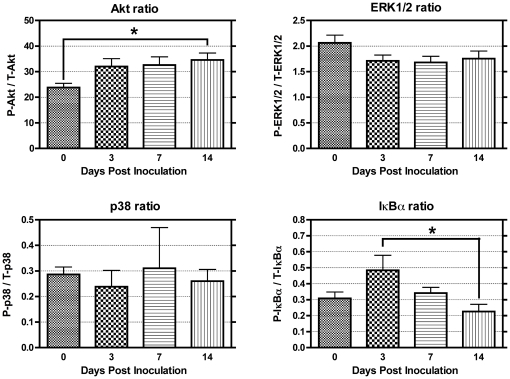
Signal transduction changes in liver due to *C. rodentium* infection. The phosphorylated/total mean fluorescence intensity (MFI) ratios were determined for liver lysates (5 µg total protein) harvested from animals over the course of *C. rodentium* infection. Average Akt activation was significantly increased at 14 DPI with comparable elevations at 3 and 7 DPI missing statistical significance. Elevations were observed in IκBα phosphorylation, particularly in mice with hepatic necrosis, demonstrating statistical significance as a group versus 14 DPI livers. ERK1/2 and p38 failed to demonstrate significant changes over the course of *C. rodentium* infection in livers (One-way ANOVA with Tukey's multiple comparison test: * P<0.05, ** P<0.01, *** P<0.001). Data presented as mean ± SEM.

To determine if systemic cytokine and chemokine profiles correlated with local levels in the liver, we analyzed lysates using 23-plex cytokine/chemokine panels and noted numerous targets that increased in serum that were also similarly upregulated in livers at 3 DPI (**Figure S4**). Liver levels of IL-1β (L), G-CSF (L), KC (L), MCP-1 (L), MIP-1α (L), and RANTES (L) were elevated at 3DPI. Given that cytokines are known signaling molecules, we also measured signal transduction in liver lysates using multiplex phospho-kits, monitoring both total and phosphorylated forms of JNK, Akt, ERK1/2, p38, IκBα, and STAT3 (phosphorylated species only) (**Figure 5**). STAT3 showed increases in livers with necrotic lesions as confirmed by western analysis (data not shown), with lower, but sustained activation at 7 and 14 DPI (**Figure 4C**). Akt activation, assessed by the ratio of phosphorylated/total Akt, was upregulated at 14 DPI (P<0.05), although levels elevated at 3 and 7 DPI did not reach statistical significance (**Figure 5**). Phosphorylation of IκBα, which results in proteasome-mediated degradation and subsequent activation of Nf-κB, was increased in animals with necrotic lesions at 3 DPI, indicating potential activation of pro-inflammatory and tissue repair mechanisms associated with this transcription factor. IκBα activation missed significance as a group when compared to 0 DPI controls but was statistically significant when compared to livers at 14 DPI (P<0.05).

### Multivariate analysis uncovers serum and tissue cytokines and chemokines that discriminate hepatic necrosis

**Figure 6:**
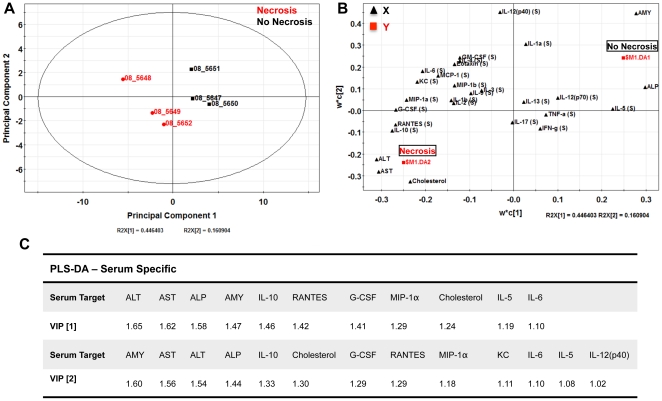
Serum-specific PLS-DA analysis of *C. rodentium* infected C57BL/6 mice at 3 DPI. (**A**) Partial least squares discriminate analysis (PLS-DA) showing separation of mice with and without necrosis using the first two principal components. (**B**) Cytokine covariation based on class discrimination using cytokine targets as independent variables (**X, black triangles**) and pathological states (presence of absence of necrotic lesions) as the dependent dummy variables (**Y, red squares**). (**C**) Variables in projection (VIPs) for principal components 1 and 2, where targets with values >1 have positive influence in discriminating between classes. Table represents the serum-specific VIPs and their respective scores that best discriminate necrotic from non-necrotic mice at 3 DPI.

**Figure 7:**
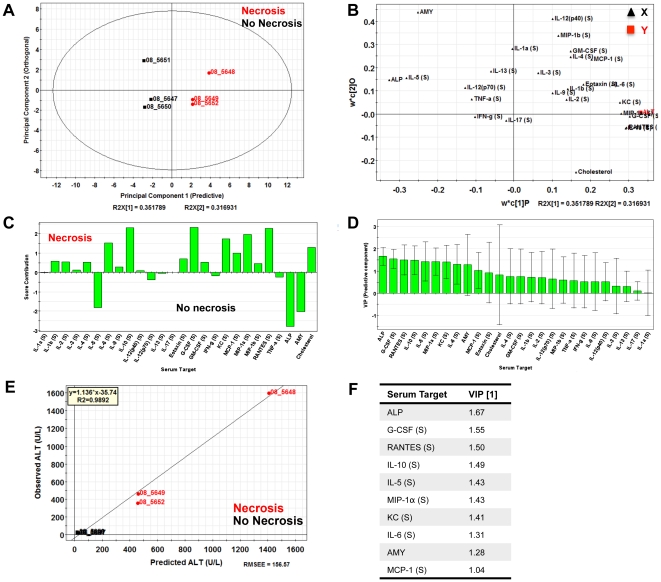
Serum-specific OPLS analysis of *C. rodentium* infected animals at 3 DPI. OPLS analysis of *C. rodentium* infected animals at 3 DPI using serum cytokines and chemistries (X) for prediction of ALT levels (Y). (**A**) Mice segregated well in the predictive component (principal component 1) with an R^2^X (1) = 0.35, indicating this component captured ∼35% of the variance present in the X variables. (**B**) The predictive weight and covariation of serum targets (**X variables,** **black triangles**) in relation to serum ALT (**Y,** **red square**). (**C**) The variables that best separate the two pathological states in relation to the predictive component. (**D**) Variables in projection (VIPs) for the predictive component where values >1 are have positive influence in determining ALT levels, and VIP <1 have less predictive influence. (**E**) Observed vs predicted plot for ALT resulted in a R^2^ = 0.9892 indicating a highly predictive model based on serum cytokines. (**F**) Table representing the VIPs for the predictive component for serum ALT.

Analysis of data based on averages in representative groups can result in oversight of important correlations noted on an individual animal basis. We therefore used a multivariate computational approach to determine features that covary best in our data compendium leveraging its multiplex nature. PLS-DA (Partial Least Squares Projection to Latent Structures - Discriminant Analysis) and OPLS (Orthogonormalized Partial Least Squares Regression) were used to determine variables with the highest discriminatory power for mice with necrotic lesions at 3DPI. Furthermore, these analyses were used to determine features that correlate best with serum ALT, a known biomarker of liver necrosis. We developed non-invasive models (serum targets only), tissue level model (liver targets only), and a combined model including both types of features. The serum PLS-DA model resulted in adequate separation of lesion bearing vs non-lesion bearing animals (**Figure 6A**), with dummy variables (Y) based on this classification covarying with serum targets that make up the principal components plane (**Figure 6B**). Their relative importance as discriminators was assessed by their variables in projection (VIPs) score for the principal component 1, where values >1 have positive influence in discriminating between classes and VIP <1 are less influential (**Figure 6C**). Similarly, using OPLS regression which reduces dimensions on the basis of their covariance with a specified dependent variable (Y, serum ALT) (principal component 1 – predictive), while ignoring targets orthogonal to this vector (principal component 2 – orthogonal) also resulted in clear separation of animals based on this classifier (**Figure 7**). PLS-DA serum models uncovered ALT, AST, immune modulators (IL-6, IL-10), monocytes chemokines/activators (MIP-1α, MCP-1), neutrophil chemokines/activators (G-CSF, KC), and T-cell activation (RANTES) as enriched in animals with hepatocellular necrosis (**Figure 6 C**).

**Figure 8:**
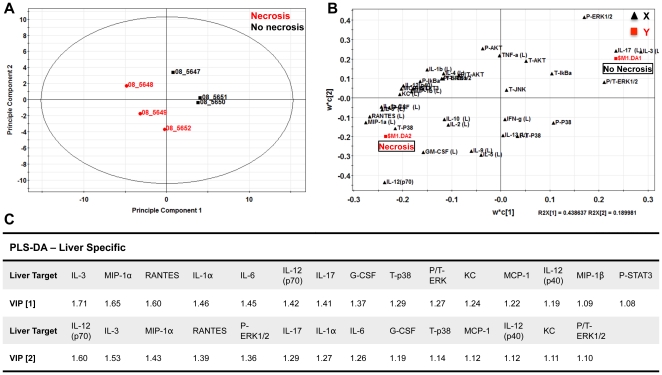
Liver-specific PLS-DA analysis of *C. rodentium* infected C57BL/6 mice at 3 DPI. (**A**) Partial least squares discriminate analysis (PLS-DA) showing separation of mice with and without necrosis using the first two principal components. (**B**) Cytokine covariation based on class discrimination using cytokine targets as independent variables (**X, black triangles**) and pathological states (presence or absence of necrotic lesions) as the dependent dummy variables (**Y, red squares**). (**C**) Variables in projection (VIPs) for the principal components 1 and 2, where values >1 have positive influence in discriminating between classes. Table represents the liver-specific VIPs and their respective scores that best discriminate necrotic from non-necrotic mice at 3 DPI.

**Figure 9:**
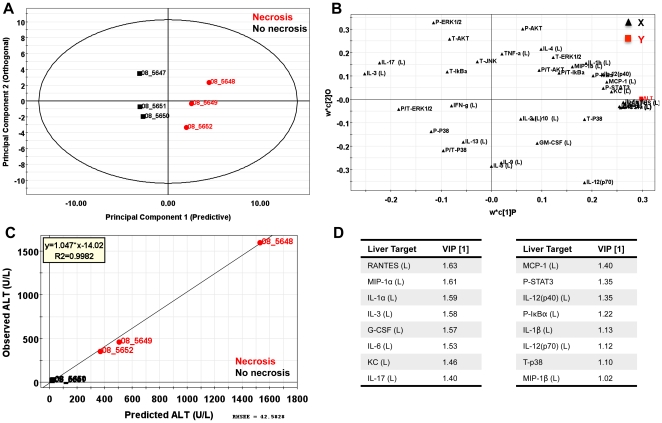
Liver-specific OPLS analysis of *C. rodentium* infected animals at 3 DPI. OPLS analysis of *C. rodentium* infected animals at 3 DPI using serum cytokines and chemistries (X) for prediction of ALT levels (Y). (**A**) Mice segregated well in the predictive component (principal component 1) with an R^2^X (1) = 0.35, indicating this component captured ∼35% of the variance present in the X variables. (**B**) The predictive weight and covariation of serum targets (**X variables,** **black triangles**) in relation to serum ALT (**Y,** **red square**). (**C**) Observed vs predicted plot for ALT resulted in a R^2^ = 0.9892 indicating a highly predictive model based on hepatic cytokines. (**D**) Table representing the VIPs >1 for the predictive component for serum ALT.

**Figure 10:**
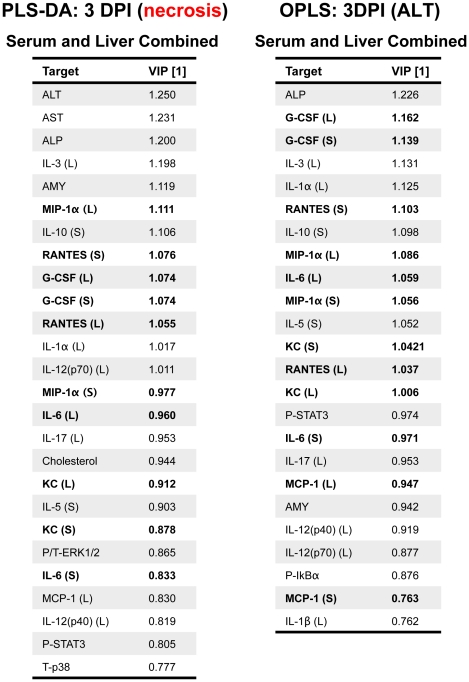
Most influential variables in serum and liver obtained from PLS-DA and OPLS models. Combined models for PLS-DA (necrosis state) and OPLS (Y = ALT) were generated using data collected on 3 DPI. Targets in bold are those that were found influential in both serum and liver.

This method was repeated for tissue markers (**Liver PLS-DA** **Figure 8** **, and OPLS** **Figure 9**) and resulted in a large overlap with the serum specific targets; immune modulators (IL-1α (L), IL-6 (L), IL-12p40 (L)), monocytes chemokines/activators (MIP-1α (L), MIP-1β (L), MCP-1 (L)), neutrophil chemokines/activators (G-CSF (L), KC (L)), and T-cell activation (RANTES (L)) as enriched in mice with hepatic necrosis. Combining both serum and liver targets for PLS-DA and OPLS models were generated and the relative VIPs compared (**Figure 10**). The results of PLS-DA and OPLS models generated using serum targets, liver targets, and combined targets are summarized in Table 1. Overall, both serum and tissue models were effective in discriminating both the presence of necrosis and it's severity. The tissue models were slightly better independently than the serum models, and the combined (serum + tissue) analysis gave the highest R^2^Y (cumulative) and Q^2^ (cumulative) with the least number of components. The non-invasive method of serum cytokine detection makes this arguably a more attractive method, even with a modest loss in model prediction.

**Table 1:** PLS-DA and OPLS component contributions for discrimination (R^2^ Y) and variance (Q^2^) of necrosis at 3 DPI.

Serum Model					
Component	R^2^Y	R^2^Y (cumulative)	Q^2^	Q^2^ (cumulative)	Model Type
1	0.789	0.789	0.441	0.441	
2	0.168	0.957	−0.336	0.385	
3	0.030	**0.987**	0.343	**0.596**	**PLS-DA: 3DPI**
1 (P)	0.842	0.842	0.547	0.547	
2 (O)	0.081	0.923	0.137	0.684	
3 (O)	0.056	**0.980**	0.198	**0.882**	**OPLS: 3DPI (ALT)**

Liver Model					
Component	R^2^Y	R^2^Y (cumulative)	Q^2^	Q^2^ (cumulative)	Model Type
1	0.735	0.735	0.492	0.492	
2	0.248	0.984	0.656	0.825	
3	0.015	**0.999**	0.860	**0.976**	**PLS-DA: 3DPI**
1 (P)	0.783	0.783	0.522	0.522	
2 (O)	0.208	0.991	0.234	0.756	
3 (O)	0.006	**0.997**	0.132	**0.888**	**OPLS: 3DPI (ALT)**

Combined Model					
Component	R^2^Y	R^2^Y (cumulative)	Q^2^	Q^2^ (cumulative)	Model Type
1+2 (27 targets)	0.983	**0.983**	0.987	**0.987**	**PLS-DA: 3DPI**
1+2 (13 targets)	0.994	**0.994**	0.976	**0.976**	**PLS-DA: 3DPI**
P+O (26 targets)	0.996	**0.996**	0.960	**0.960**	**OPLS: 3DPI (ALT**)

Model results are indicated based on using serum or tissue targets alone as well as combined models using the top variables from each independent model.

## Discussion

In the present study we characterized the systemic and liver effects of *C. rodentium* infection in female C57BL/6 mice at early, peak, and resolving timepoints of bacterial clearance. We demonstrated systemic targets (cytokines/chemokines and serum chemistry markers) that differentiated mice with and without tissue changes by PLS-DA. Systemic elevations in ALT, AST, with an upregulation of immune modulators (IL-6, IL-10), monocytes chemokines/activators (MIP-1α, MCP-1), neutrophil chemokines/activators (G-CSF, KC), and T-cell activation (RANTES) at 3 DPI correlated with coagulative liver necrosis, with a predominant periportal distribution in association with venous (portal) fibrin thrombi. Mice harboring liver lesions also demonstrated induction of STAT3 and IκBα phosphorylation, coupled with liver specific protein elevations of IL-1β (L), IL-6 (L) G-CSF (L), KC (L), IL-12p40 (L), MCP-1 (L), MIP-1α (L), and RANTES (L). These changes occurred prior to peak *C. rodentium* colonization in the absence of significant co-existing colonic lesions and disease.

Pathological assessment of livers indicated that 50% of mice at 3 DPI had periportal necrotic lesions associated with portal vein fibrin thrombi that have not been observed and/or previously reported in this murine model. These results have been repeated in our lab in subsequent studies (unpublished results). This may be due in part to the fact that 3 DPI is early in colonic disease pathogenesis coupled by our interest in investigating an organ distal to the primary site of disease pathology and bacterial colonization. Bacterial translocation due to inflammation or overgrowth of commensal bacteria has been shown to occur, with translocation of luminal bacteria to other organs [21], [22], in some cases resulting in sepsis and the subsequent death. Furthermore, *C. rodentium* has demonstrated translocation to MLNs during infection course [23]. We did not culture liver tissues to determine levels of *C. rodentium* colonization but follow-up experiments in which liver sections are stained using a *C. rodentium*-specific antibody could confirm hepatic colonization. *C. rodentium* has been associated with disruption of tight junctions and barrier function in intestinal epithelial cells *in vitro* and *in vivo* [24], [25]. Given liver lesions were predominantly periportal in their pattern of distribution, and indicative of thrombotic ischemic injury with the presence of portal vein thrombi, it is likely that bacterial components, or *C. rodentium* (live or dead) at the early stages of bacterial colonization, translocated from the gut lumen via portal blood to the liver inducing systemic and hepatic inflammatory processes. Lipopolysaccharide (LPS), for example, through interaction with LPS-binding protein and TLR4 has been demonstrated to induce fibrin clots in tissues [26]. LPS has also been shown to induce expression of ATP-binding cassette transporter A1 (ABCA1), a transporter known to promote apolipoprotein-dependent cholesterol efflux from cells, with cholesterol participating in the removal of LPS [27], which could contribute to elevated levels of cholesterol noted in our study. Alternatively, it is also possible that at early stages in the pathogenesis of *C. rodentium*, coagulative factors such as PAI-1 may be altered. These factors have been associated with increased portal vein thrombosis (PVT) in patients with liver diseases [28] and increased fibrin deposition in LPS models of idiosyncratic liver injury [29], warranting future analysis of hemostatic factors in this model. It has been proposed that the liver may act to trap LPS, along with other bacterial components as a protective mechanism to prevent systemic spread of bacteria. As a regenerative organ, minimal necrosis via coagulative mechanisms may be manageable in the liver, and preferable to dissemination of these products to other organs and generating a systemic inflammatory response. The presence of necro-granulomatous inflammation at later timepoints indicates residual effects of hepatic mechanisms to repair prior hepatic injury in these animals. The lack of increased circulating ALT indicates that active necrosis was likely not occurring at 7 and 14 DPI, indicating transient injury.

Using PLS-DA and OPLS modeling, we demonstrate an upregulation of targets associated with necrosis at 3 DPI (for total list see **Figure 10**). G-CSF (S/L), MCP-1 (S), KC (S/L), IL-6 (S/L), IL-10 (S), and RANTES (S/L) were highly predictive for animals with hepatocellular necrosis and demonstrated high covariation with ALT. Neutrophil activation is commonly observed at the site of tissue injury. Indeed, G-CSF, a chemokine known for neutrophil recruitment and activation, was elevated in serum and liver homogenates. G-CSF is also known to promote the survival, proliferation, differentiation, and function of both precursor and mature neutrophils. The increased levels of G-CSF may also account for the induction of STAT3 and Akt levels found in the liver. Interestingly, IL-6 can inhibit the inflammatory response induced by neutrophil-activating chemokines by facilitating neutrophils apoptotic clearance [30], [31]. Mechanistically, this has been demonstrated to be a result of STAT-3 activation in a model of acute peritoneal infection [32]. Deregulation of gp130 (IL-6 receptor) signaling in this model did not affect the initial CXCL1/KC-driven neutrophil recruitment, indicating early induction of these chemokines is IL-6 independent [32]. IL-10 (S) was also elevated in necrotic bearing mice in our model, a cytokine with known hepatoprotective affects in drug-induced liver injury. IL-10 can regulate KC levels *in vivo* attributable to modulation of STAT 1 and STAT3 (non-gp130 mediated) signaling [33], as well as iNOS levels in an acetaminophen-induced model of liver injury [34], [35].

*C. rodentium* also induced cytokines MCP-1 and MIP-1α in mice with hepatic necrosis, these cytokines are implicated in the process of hepatic inflammation and are known to recruit monocytes and lymphocytes during liver injury. MCP-1 is primarily secreted by monocytes, macrophages, and dendritic cells. It activates macrophages and has chemotactic activity for monocytes [36] and basophils but not for neutrophils or eosinophils. MCP-1 is also associated with numerous models of fibrosis, and has been shown to activate hepatic stellate cells, which play a major role in hepatic fibrosis. In the current study, the occurrence of portal vein thrombi in livers with hepatic necrosis, paralleled increases in serum and liver MCP-1 demonstrated by PLS-DA. Analysis at 3 DPI also showed covariation with P-IκBα; a known regulator of Nf-κB transcription. P-IκBα has also shown to function in an autocrine loop with RANTES, and demonstrated a high correlation with MCP-1 (R^2^ = 0.84) in our study (**Figure S5** comparing 3 and 7 DPI networks). Collectively, the regulatory control by both STAT and Nf-κB mediated transcription deserve further exploration of their precise roles in mediating the responses to early infection and liver inflammation and injury in this model.

Liver responses detected in infected animals at 3 DPI overlapped many targets associated with acute colitis and disease severity including MCP-1, MIP-1α, MIP-1β, RANTES, and KC and neutrophil/Th17-related targets such as KC, IL-1β, IL-6, IL-12/23p40, and G-CSF. Local responses in the liver to intestinal stimuli are a substantial source of circulating cytokines [37]. Portal circulation potentially containing bacterial components and/or cytokines and chemokines drains the intestines before entering the systemic circulation [38]. It is possible that these cytokine responses are generalized systemic responses to the presence of bacterial products, but may also indicate a specific liver response to distal infection in the gut. Indispensable for metabolic processes, the liver is also a key “immunologic” organ [8]. Hepatic perfusion with venous blood return from the gastrointestinal tract highly enriched with microbial antigens provides early host defense against microbes and toxins translocating across the gut wall [7]. Recently, severe necrotizing hepatitis was observed in IL-10 KO mice orally infected with the enteric parasite *Trichinella spiralis* [39]. Douglas *et al* demonstrated the critical role of IL-10 to mediate the trafficking of intestinally derived CD4^+^ T-cells expressing the IL-4 cytokine required for neutrophil-dependent necrosis. Sequestration of activated neutrophils was shown to be dependent on IL-4, and while neutrophil depletion alleviated necrosis, this was not required for initiation of injury. Thus, enterohepatic cytokine balance is important for appropriate hepatic immune function. Serum IL-10 was induced in *C. rodentium*-infected animals at 3 DPI and associated with coagulative necrosis. A regulatory cytokine, IL-10, exerts influence on numerous immunological activities such as antigen presentation and cytokine production, and is involved in the initiation and maintenance of inflammation [40]. Induction of intestinal inflammation using dextran sulfate sodium (DSS) during experimental NASH promotes translocation of LPS, hepatic inflammation, and fibrogenesis [41]. *Helicobacter hepaticus (H. hepaticus)*, another enteric pathogen known to induce spontaneous, chronic colitis in certain susceptible mouse strains, is also associated with active chronic hepatitis and development of hepatocellular carcinoma in both inbred and knockout strains of mice [42], [43], [44]. *H. hepaticus* is now widely used to examine the role of intestinal microbiota on host inflammatory responses. Consistent with our findings, these examples collectively demonstrate a balance within the gut-liver axis appears critical in mediating host-pathogen equilibrium, a failure of which can result in detrimental consequences in both intestinal, liver, and systemic health [38], [45]. The source or stimuli for local and systemic changes in cytokines and chemokines remains uncertain. Systemic cytokine levels correlate with observed pathologies determined by PLS-based modeling in a complex data and also predictive for known biomarkers of injury such as serum ALT for liver necrosis. While it is outside the scope of this study to definitively characterize the source (colon or liver) and cell type responsible for circulating cytokines and chemokines observed at 3 and 7 DPI, this is an active area of research and future studies will hopefully clarify our understanding of the pathogenesis of hepatocellular necrosis in this model.

Additional studies in our laboratory examining liver injury at early stages of *C. rodentium* pathogenesis have confirmed that acute hepatic injury in this model is reproducible (unpublished results). Specifically, the incidence of liver necrosis at 3 DPI was increased to 5/6 animals following an overnight fast prior to necropsy, with an increased hepatitis index noted in the absence of hepatocellular necrotic lesions by 7 DPI. Timepoint controls demonstrated no observable liver necrosis or appreciable elevations in ALT, indicating that liver lesions are infection-induced and not a result of animal handling or stress. To better understand the temporal kinetics of injury, collecting serial bleeds from animals preceding, during, and under conditions of resolving liver injury could be insightful towards understanding the kinetics of cytokine production and whether these biomarkers of inflammation are precursors to injury or simply a consequence. Furthermore, use of specific neutralizing antibodies or conditional knockout studies may help to further elucidate the importance of these markers in the predisposition and severity of liver injury and homeostasis.

In conclusion, we show for the first time distinct liver pathology associated with enteric infection with *C. rodentium* in C57BL/6 mice, characterized by increased inflammation and hepatitis index scores as well as prominent periportal hepatocellular coagulative necrosis indicative of thrombotic ischemic injury during the early course of *C. rodentium* pathogenesis. Histologic changes in the liver correlate with serum elevation of liver transaminases, systemic and hepatic cytokine and chemokine changes, as well as signal transduction changes prior to peak bacterial colonization and colonic disease. *C. rodentium* infection in C57BL/6 mice appears to be a useful new model to study acute liver injury and inflammatory stress under conditions of gastrointestinal infection analogous to enteropathogenic *E. coli* infection in humans.

## Materials and Methods

### Ethics Statement

The Institutional Animal Care and Use Committee (IACUC) at the Massachusetts Institute of Technology (MIT) (0207-020-10) approved all animal experiments described.

### Mouse Infections

24 female 8–10 week old (∼20 gram) C57BL/6J (The Jackson Laboratory, Bar Harbor, ME) mice were housed six per microisolator cage in a specific pathogen-free facility approved by the Association for Assessment and Accreditation of Laboratory Animal Care. Animals were maintained on pelleted rodent chow (LabDiet, Purina Mills, Inc., Richmond, IN), water *ad libitum* and allowed to acclimate one week prior to experimentation. Infectious colitis was induced by intragastric inoculation with ∼1×10^9^ CFU of *C. rodentium* (DBS120) in 3% sodium bicarbonate (w/v in 1× phosphate buffered saline (PBS), pH 7.4), 100 µL volume as described previously [46], while the uninoculated control groups received 100 µL of 3% sodium bicarbonate vehicle. Control animals (0 DPI) were euthanized within 1 hr of inoculation and infection kinetics (3, 7, 14 DPI animals) was monitored by fecal shedding (3, 7, 10, 14 DPI) determined by plating serial 10 dilutions of fecal slurries (10% [w/v] in 1× PBS, pH 7.4) on LB agar with selection for kanamycin. At their respective timepoints, animals were euthanized by CO_2_ asphyxiation, blood collected by terminal cardiac puncture for serum chemistries and cytokines analysis (stored at −80°C until processing), and animals necropsied for tissue collection (flash frozen in liquid nitrogen, and stored at −80°C until processing).

### Histopathology

Colons and livers were formalin-fixed (10%), paraffin-embedded, and 5 µ sections stained by H&E for histological assessment by a board certified veterinary pathologist blinded to study treatment groups. Disease severity of colonic sections was based on an existent method assessing inflammation, edema, epithelial defects, crypt atrophy, hyperplasia, and dysplasia and graded on a scale from 0 to 4. Livers were assessed for both inflammatory and necrotic parameters. Degree of hepatic inflammation was graded on a scale from 0 to 4 based on region (lobular, portal, and interface), and the number of lobes with ≥5 inflammatory foci was noted. The summation of these categorical inflammatory scores resulted in a hepatitis index; mice with a score ≥4 were defined as having hepatitis.

A modified scoring criterion was developed to define the extent, degree, and pattern of liver necrosis. The overall degree or grade of necrosis was scored from 0 to 4 on the basis of the severity and distribution of the necrotic lesions and the number of lobes affected as follows: 0 – None, 1 – Minimal to Mild, 2 – Moderate, 3 – Marked, 4 – Severe to Diffuse. To aid the overall grading the distribution of necrosis was evaluated as follows: 0 – None, 1 – Focal, 2 – Multifocal, 3 – Translobular, 4 – Submassive to Massive, involving multiple lobules or the entire lobe or multiple lobes. The histological pattern of liver injury was evaluated as centrilobular, midzonal, or periportal in nature (0 = none, 1# = low, 2 ## = medium, 3 ### = high).

### Immunohistochemistry

Formalin-fixed paraffin-embedded livers and intestinal sections were stained to assess the degree of proliferation (Ki-67) and apoptosis (activated caspase 3). Anti-activated caspase 3 (Cell Signaling Technologies, Inc., Beverly, MA) and anti-Ki-67 antibodies were used as specified by the manufacturer. Antibodies were detected with biotinylated goat anti-rabbit IgG (Sigma-Aldrich, St. Louis, MO) and sections visualized with diaminobenzidine and hematoxylin counterstained. For quantification of Ki-67 labeling index, the average number of KI-67^+^ cells/mm^2^ liver determined in 15 fields (magnification, 400×, 3 fields/lobe) per mouse (*n* = 3 for 0, 7, and 14 DPI; *n* = 2 for 3 DPI (necrosis bearing); *n* = 1 for 3 DPI (no necrosis)). Data was represented as box-whisker plots, where boxes represent the first to third quartile and a horizontal line indicates the median. Bars represent ranges. Groups were compared by one-way ANOVA with Tukey's multiple comparison test.

### Clinical Chemistries

Serum samples were thawed on ice and diluted 1∶4 in sterile ddH_2_0 and processed on an Olympus AU 400e serum chemistry analyzer (Beckman Coulter, Inc., Brea, CA) for 18 serum chemistry targets. All samples were run against internal standards and machine calibrated prior to use as specified by the manufacturer.

### Multiplex Detection of Serum Cytokines and Chemokines

Mouse serum collected at necropsy was processed using mouse 23-plex cytokine panels (Bio-Rad, Hercules, CA) as specified by the manufacturer. Briefly, serum was diluted in species-specific sample diluent (1∶4) and 50 µL of sample or premixed standards were incubated with pre-washed target capture antibody-conjugated microspheres provided and incubated for 30 minutes with orbital shaking (300 rpm) in a 96-well plate. Upon washing, beads were incubated with detection antibody (30 min), washed, and subsequently incubated with streptavidin-PE (10 min). Beads were washed and resuspended with 125 µL assay buffer and read on the Luminex 200 suspension array system using the low RP1 target setting (High PMT) for maximum sensitivity. Data analysis was carried out with the Bio-Plex Manager™ 5.0 software and cytokine or chemokine concentrations calculated against an 8 point standard curve generated by either 4PL or 5PL curve fitting.

### Multiplex Detection of Liver Cytokines, Chemokines, and Phospho-Signaling

Colon and livers were thawed on ice and ∼30–50 mg of liver was briefly washed with 500 µL ice-cold cell wash buffer (Bio-Rad, Hercules, CA). Livers were then transferred to a clean pre-weighed eppendorf tube and resuspended 12× the tissue weight with cell lysis buffer (Bio-Rad, Hercules, CA). Tissues were homogenized on ice for 1 minute with a Tissue Tearor™ and subsequently frozen to −80°C overnight. Tissues were then thawed on ice and sonicated on level 5 with 5 short (∼3 sec) bursts. Upon sonication, lysates were centrifuged for 10 minutes (5,000 rpm, 4°C). Resultant supernatant was carefully removed, protein quantified by BCA protein assay (Thermo Scientific, Rockford, IL), and adjusted to 1 µg/µL with 1× PBS + 0.5% BSA (w/v). 50 µL of adjusted lysate was subsequently loaded on to a Mouse Group I: 23-Plex panel (Bio-Rad, Hercules, CA) and standards resuspended in the appropriate matrix. Samples were subsequently processed as specified above for serum cytokines and chemokines analysis using the Bio-Plex array system and software manager.

### Immunoblot of Phospho-proteins

Tissues were thawed on ice, suspended in Bio-Rad cell lysis buffer with provided phosphatase and protease inhibitors, and homogenized with a Tissue Tearor™ for 1 minute. Upon freeze thawing (−80°C), samples were sonicated on level 5 for three 10 second bursts, incubated on ice for 20 minutes, and centrifuged at 4°C at 5000 rpm for 15 minutes to precipitate insolubles. Supernatants were protein quantified by BCA (Thermo Scientific, Rockford, IL) and adjusted to 2 µg/µL with Bio-Rad lysis buffer. Equal volumes of 2× SDS loading buffer was added and 20 µL loaded (20 µg/well) onto 10% SDS Tris-glycine gels (Invitrogen, Carlsbad, CA). Upon transfer to immobolin^PQ^ PVDF membranes, blots were incubated with antibodies (1∶2000 P-STAT-3, 1∶2000 anti-rabbit monoclonal (Cell Signaling Technologies, Danvers, MA) in TBST-5%BSA, incubated with Santa Cruz luminal reagent, exposed by Kodak MR film, and quantified by NIH imageJ.

### PLS-DA and OPLS Analysis

The data was mean centered, variance scaled, log_10_ transformed, and analyzed by multivariate analysis using SIMCA-P 11.5 software (Umetrics Inc., Kinnelon, NJ). PCA analysis was used to reduce the dimensionality of the data set and to assess the covariation of variance across measured targets. PLS-DA analysis was used to determine the features that best differentiated between selected groups of animals designated as classes (i.e., mice with or without hepatocellular necrosis). OPLS analysis was used to determine the features that best correlated with a desired Y variable, such as ALT levels or degree of necrosis/hepatitis, resulting in variable importance in the projection (VIP). VIPs greater ≥1 were considered significant in their contribution to the model predicting the dependent variable (Y) of interest. Model quality was assessed and reported using the following parameters: R^2^ Y, the fraction of the sum of squares of all Y variables explained by the component of the model, and R^2^ Y cumulative, the cumulative sum of squares of all Y variables explained by all components of the model. Q^2^ is the fraction of the total variation in Y variables that can be predicted by the component, and Q^2^ cumulative is the cumulative Q^2^ of the Y variables for all components in the model. An R^2^ cumulative and Q^2^ cumulative of 1 indicate a perfect fit, with 100% of the relationship between X variables and Y variables explained.

### Statistical Analysis

Statistical significance in body weight change, bacterial load, disease indices, cytokine changes, clinical chemistries, and phosphorylation levels was determined by comparing control or experimental groups by either student's T-test or by two-way analysis of variance (ANOVA) followed by Tukey's post tests. Data demonstrating non-normal distributions or categorical in nature (i.e., pathology scores) were assessed by Kruskal-Wallis non-parametric test with Dunn's multiple comparison test against controls unless otherwise indicated. If only two groups were analyzed, Mann Whitney was utilized. All analyses were done using GraphPad Prism software version 4.0 (La Jolla, CA), and *P* values of <0.05 were considered significant.

## Supporting Information

Figure S1 **Infection kinetics and body weight changes in C57BL/6 mice infected with** ***C. rodentium.*** *C. rodentium* bacterial burden (**A**) was detectable by 3DPI and maximal around 7 DPI with clearance beginning by 10 DPI. Monitoring of body weights (**B**) demonstrated no statistically significant weight loss over the course of infection.
(TIF)

Figure S2 **Serum chemistry changes in C57BL/6 mice inoculated with** ***C. rodentium*** **.** Systemic parameters assessing liver function (ALT, AST, ALP, total bilirubin), kidney function (creatinine, BUN, CPK) and electrolytes (Ca^2+^, Cl^−^, Na^+^, K^+^) were measured at 0, 3, 7, and 14 DPI. (One-way ANOVA with Tukey's multiple comparison test: * P<0.05, ** P<0.01, *** P<0.001). Lines indicate group means.
(TIF)

Figure S3 **Systemic cytokine and chemokine changes induced by** ***C. rodentium*** **infection in C57BL/6 mice.** Serum cytokines and chemokines were measured by quantitative multiplex analysis using Luminex technology. (One-way ANOVA with Tukey's multiple comparison test: * P<0.05, ** P<0.01, *** P<0.001). Lines indicate group means (*n* = 5 to 6 animals per timepoint).
(TIF)

Figure S4 **Liver cytokine and chemokine changes induced by** ***C. rodentium*** **infection in C57BL/6 mice.** Liver cytokines and chemokines were measured by quantitative multiplex analysis using Luminex technology. Statistically significant changes as a group were only found for IL-1β (L), MCP-1 (L), MIP-1**α** (L), and RANTES (L) (One-way ANOVA with Dunnett's multiple comparison test comparing all columns to controls: * P<0.05, ** P<0.01, *** P<0.001). Lines indicate group means (*n* = 6 livers per timepoint).
(TIF)

Figure S5 **PLS-DA analysis of 3 and 7 DPI animals.** Animals were assigned one of three classes; 3DPI (no necrosis), 3 DPI (necrosis), and 7 DPI. (**A**) Animal separation based on the first two principal components. (**B**) Target covariation using all serum target, liver targets, and histological scores.
(TIF)
