# Supplementary figures and images for: Enteric Infection with Citrobacter rodentium Induces Coagulative Liver Necrosis and Hepatic Inflammation Prior to Peak Infection and Colonic Disease
Source: PLoS One. 2012 Mar 9;7(3):e33099. doi: 10.1371/journal.pone.0033099 (PMC3302869; doi:10.1371/journal.pone.0033099)

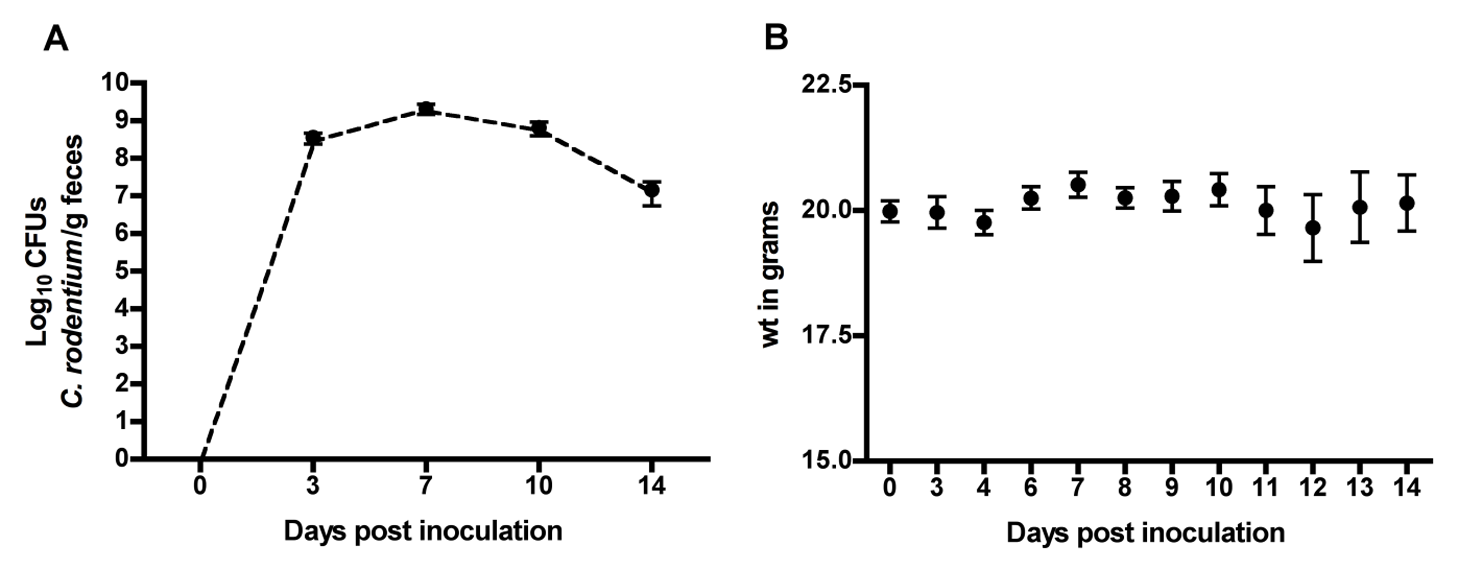

Supplement: Figure S1 — Infection kinetics and body weight changes in C57BL/6 mice infected with C. rodentium. C. rodentium bacterial burden (A) was detectable by 3DPI and maximal around 7 DPI with clearance beginning by 10 DPI. Monitoring of body weights (B) demonstrated no statistically significant weight loss over the course of infection. (TIF) [file pone.0033099.s001.tif]

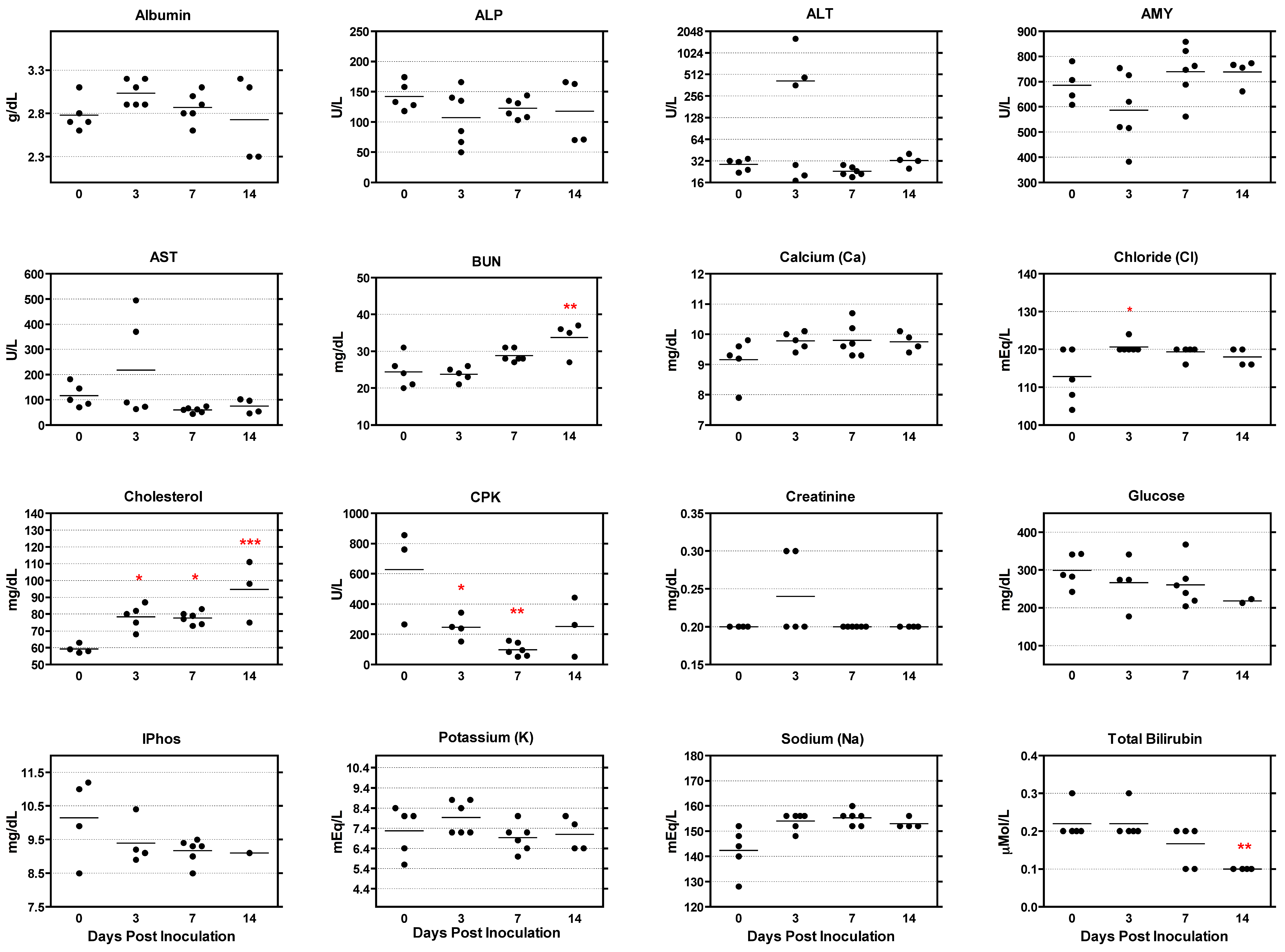

Supplement: Figure S2 — Serum chemistry changes in C57BL/6 mice inoculated with C. rodentium . Systemic parameters assessing liver function (ALT, AST, ALP, total bilirubin), kidney function (creatinine, BUN, CPK) and electrolytes (Ca2+, Cl−, Na+, K+) were measured at 0, 3, 7, and 14 DPI. (One-way ANOVA with Tukey's multiple comparison test: * P<0.05, ** P<0.01, *** P<0.001). Lines indicate group means. (TIF) [file pone.0033099.s002.tif]

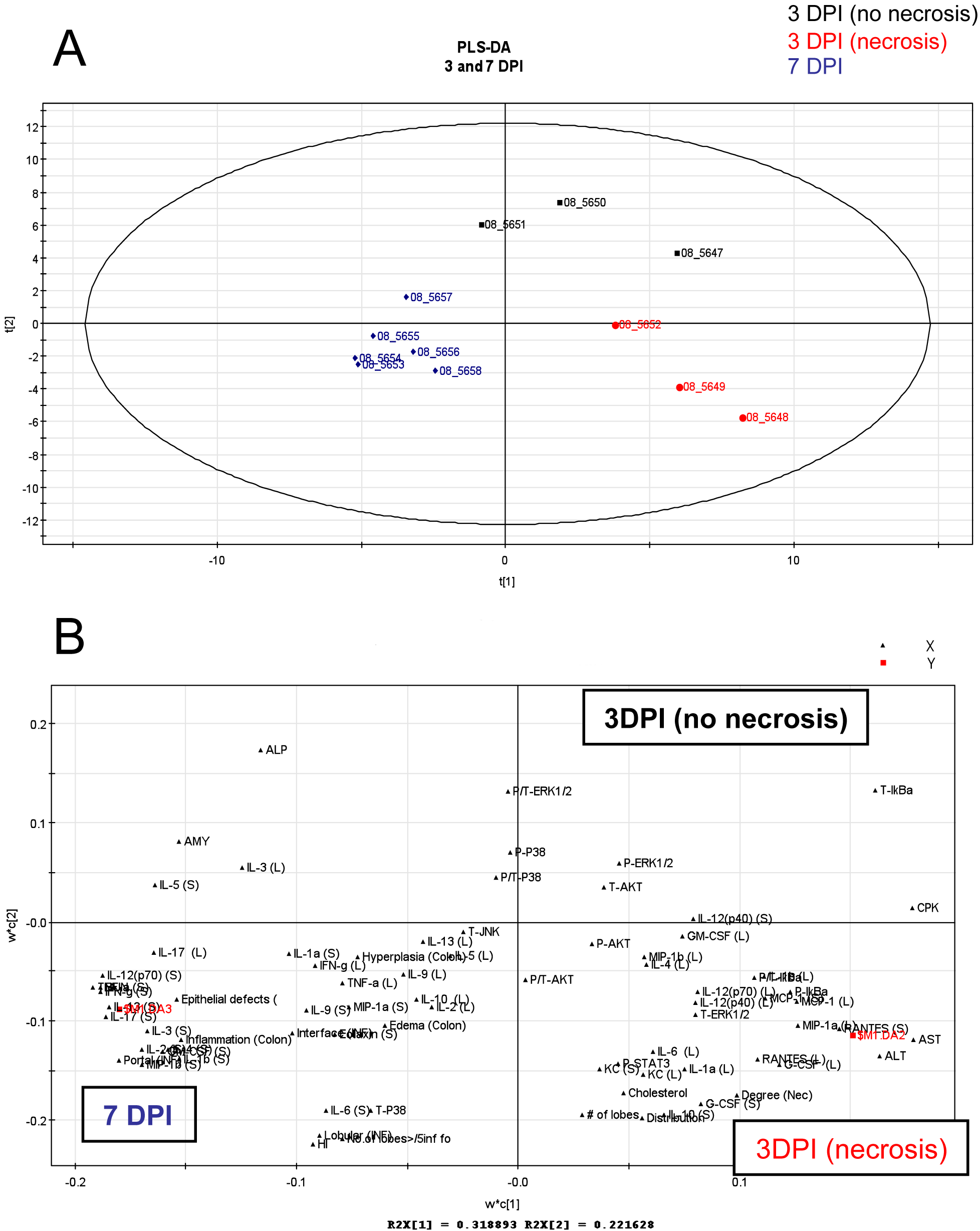

Supplement: Figure S5 — PLS-DA analysis of 3 and 7 DPI animals. Animals were assigned one of three classes; 3DPI (no necrosis), 3 DPI (necrosis), and 7 DPI. (A) Animal separation based on the first two principal components. (B) Target covariation using all serum target, liver targets, and histological scores. (TIF) [file pone.0033099.s005.tif]
